# A critical review of consumer responsibility in promoting sustainable cocoa production

**DOI:** 10.1016/j.crfs.2024.100818

**Published:** 2024-08-10

**Authors:** Shahida Anusha Siddiqui, Ikawati Karim, Chardi Shahiya, Sergey Shityakov, Widya Satya Nugraha, Gyula Kasza

**Affiliations:** a Independent Researcher, Germany; b Agribusiness Department, Universitas Sulawesi Barat, Majene, 90311, Indonesia; c LInfochemistry Scientific Center, ITMO University, Saint Petersburg, 191002, Russia; d Laboratory of Chemoinformatics, Infochemistry Scientific Center, ITMO University, Saint-Petersburg, Russia; e Department of Agricultural Socio-Economics, Faculty of Agriculture, Universitas Gadjah Mada, Yogyakarta, Special Region of Yogyakarta, 55281, Indonesia; f Doctoral School of Food Science, Hungarian University of Agriculture and Life Sciences, Vill ´anyi Street 29-43, 1118, Budapest, Hungary; g Institute of Food Chain Science, University of Veterinary Medicine Budapest, H-1078, Budapest, István utca 2., Hungary

**Keywords:** Consumer buying behavior, Life cycle assessment (LCA), Production chain, Sustainable product

## Abstract

Consumer buying behavior can be defined as all the different steps that consumers follow before purchasing a good or service. Web browser research, involvement in online networking discussions, and a range of other activities might be a part of this process. Despite the negative effects of its production chain on the environment, and on the socio-economical condition of local farmers, chocolate products are among the most distributed food and beverage items in the world. In this review, the consumer responsibility for sustainable cocoa production is described. This study determines the consumer opinions and attitudes on the different operations pursued in the production chain of chocolate, from the collection of cocoa beans to their processing into different final products. For this, data on life cycle assessment from some studies was gathered to identify and investigate links between the production chain of different types of chocolate (dark, white, milk) and its impact on natural resources so that the sensitivity of consumers to purchase more sustainable products can be evaluated. This approach revealed that consumers will not only purchase chocolate because of its good quality or health benefits, but they also consider it the most sustainable product.

## Highlights

• Production process, knowledge, preference, product qualities, economic and socio-demographic variables all have a role in shaping consumers' intentions to buy and behaviors about cocoa and cholate production.
• The cocoa production process is linked to several environmental issues, such as the emissions of greenhouse gas, deforestation, water pollution, waste generation, and soil erosion; certifications and labeling were shown to have a significant impact on the promotion of environmentally conscious cocoa production.
• Packaging material sustainability was discovered to be a significant aspect of promoting sustainable chocolate production. Life cycle methodology can help determine the sustainability of chocolate in its production process.

## Graphical abstract

**Figure:**
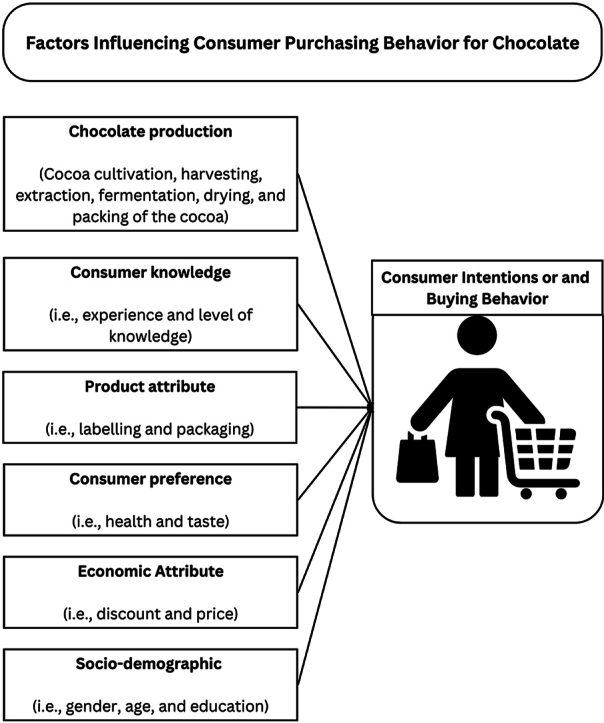

## 1 Introduction

From cocoa beans to final products such as chocolate and other derivative products is a long supply chain process, whereas these products are favored by consumers both in local and global markets; hence, the sustainability aspect needs to be considered in environmental, economic, and social aspects. This product's very high business potential is necessary to understand better consumer behavior (Del Prete and Samoggia, 2020a). In this case, consumer purchasing in the food industry is caused by various factors such as people's recommendations, brand, price, personal experience, health restrictions, and allergies. The other factors are flavor, quality of nutrition, nation of origin, and packaging (Kozelová et al., 2014). Moreover, many consumers believe that purchasing and consuming chocolate should pay attention to sustainability and ethical consumption issues (Kutaula and Gillani, 2018). This is due to rising awareness of the social and environmental implications involved with chocolate production (Konstantas et al., 2018) (see Table 1).

**Table 1:** The leading exporter and main manufacturer of chocolate worldwide.

No	Leading exporter		Chocolate manufacturing	
	Country	Trade value (US$ billions)	Company	Net sales (US$ millions)
1	Germany	5.64	Mars Wrigley Confectionery, division of Mars Inc (USA)	20,000
2	Belgium	2.8	Ferraro Group (Italy)	13,566
3	Italy	2.44	Mondelez International (USA)	11,467
4	Poland	2.36	Hershey Co (USA)	8066
5	Netherlands	2.25	Nestle SA (Switzerland)	7636
6	Canada	1.97	Chocoladenfabriken Lindt & Sprungli AG (Switzerland)	4574
7	US	1.74	Pladis (UK)	4655
8	UK	1.01	Lindt & Sprungli AG	4331
9	Switzerland	0.88	Ezaki Glico Co. Ltd.	3311
10	Mexico	0.66	–	–

**Fig. 1:**
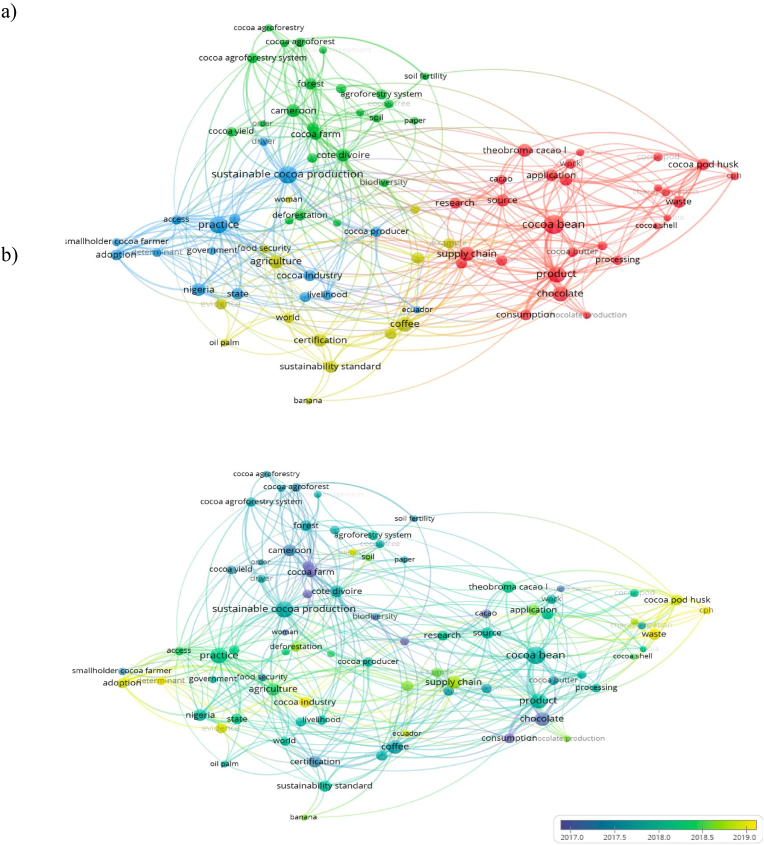
A bibliometric network map of scientific research on sustainable cocoa production. A network visualization based on four color clusters a) indicates the relatedness of scientific journal articles and overlay visualization, b) specifies a period of occurrence of the keyword from 2017 (blue) to 2019 (yellow). These figures are created from VOSviewer and data were collected from Scopus database, Web Science, and other updated scientific sources.

Moreover, Fig. 1 indicates the numerous study subject trends associated with cocoa production. Nowadays, cocoa sustainability faces some obstacles, both internal and external, that restrict development toward environmentally friendly and socially responsible approaches. Internal challenges encompass several issues, such as the use of monoculture farming, insufficient technical assistance for tree planting, nutrient competition between shade trees and cocoa trees, and inadequate land tenure policies (Grant et al., 2022; Olutegbe and Sanni, 2021). These variables also lead to decreased production, environmental deterioration, and limited sustainability in cocoa farming. External factors such as limited understanding and institutional support are other variables that might inhibit the achievement of sustainability in the cocoa sector. The current major challenge in cocoa production is climate change, which results in unpredictable weather. According to Oyekale (2020), farmers faced significant difficulties due to extremely high temperatures or stormy rainfall, which affected the quantity and quality of cocoa beans.

The growing number of cocoa production issues is worsened by the worldwide market requiring a huge quantity of cocoa beans. According to Future Market Insights (2023), the worldwide market for cocoa beans in 2023 is expected to be US$ 13,603.2 million, with Germany accounting for around 17.6% of the European market. These challenges highlight the complexity of achieving sustainability in the cocoa industry by affecting the cocoa sector's economic feasibility, environmental effects, and social aspects.

In the European market, the industry for cocoa beans requires sustainability in a supply chain with high-quality attributes of cocoa beans to fulfill a variety of end users. In this concurrent world, the quality of cocoa beans is important to meet market demands for cocoa-derived products as well as chocolate products.

In this case, the variables of food safety, efficiency, effective cost, taste, and high quality of cocoa beans for consumer demands play an important role in the cocoa supply chain and sustainability. Additionally, the European market determines the quality standard of cocoa beans through international food safety standards. The most important thing is the nutritional and flavor components. Like chocolate demands, it is a highly competitive market, and the critical point is about the high quality of cocoa beans as a basic material. One of the major companies for chocolate as well as cocoa processors is Barry Callebaut.

In 2022, Germany emerged as the leading global exporter of chocolate, with exports exceeding $5.6 billion in value. Over the past decade, there has been a consistent upward trend in Germany's chocolate exports. In 2010, the volume of exports amounted to around 667,000 metric tons. However, by 2020, exports significantly increased, reaching over one million metric tons. Conversely, the United Kingdom (UK) experienced a significant increase in chocolate imports over the last two decades. The import value of chocolate has seen more than a fourfold increase from around 400 million British Pounds in 2001 to roughly 1.75 billion British pounds in 2020. Notably, imports from EU countries have steadily risen while those from non-EU countries have remained relatively minor. However, there has been a slight decline in the sales volume of domestically manufactured chocolate within the UK over recent years (Statista, 2023).

Despite encountering several obstacles along the whole food supply chain, including the socioeconomic state of local farmers and environmental concerns, chocolate products continue to be highly consumed globally. However, it has been observed that consumers play a pivotal role in influencing the production of chocolate in a more sustainable manner. In addition, demand for more sustainably produced chocolate is anticipated to rise in tandem with the growing concerns around sustainability. Increased consumer demand and pressure might potentially speed the development of a sustainable cocoa production system. Therefore, the primary objective of this research is to provide a comprehensive and in-depth examination of consumers' role in promoting sustainable cocoa production.

## 2 Methodology

The Preferred Reporting Items for Systematic Reviews and Meta-Analyses (PRISMA) standards were followed for conducting this systematic review (Adli et al., 2022). The review includes academic research on consumer responsibility in promoting sustainable cocoa production. Keywords were chosen in order to search articles in the following reputable and authoritative research databases, such as the Scopus database. Boolean moderators (i.e., "AND" and "OR") and quotation marks were used to find the relevant and appropriate literature. Additionally, the keywords that this study used were "consumer responsbility" OR "consumer preference" OR "consumer behaviour" AND "sustainable cocoa production" OR "cocoa production" OR "cocoa".

Furthermore, researchers found 54 articles in the Scopus database. The initial selection focused on articles published after they were peer-reviewed and written in English that addressed consumer responsibility in promoting sustainable cocoa production or sustainable cocoa production itself. Titles, abstracts, and full texts of these articles were then reviewed. Articles not aligned with the topic were excluded, resulting in a second selection of 20 relevant papers.

**Fig. 2:**
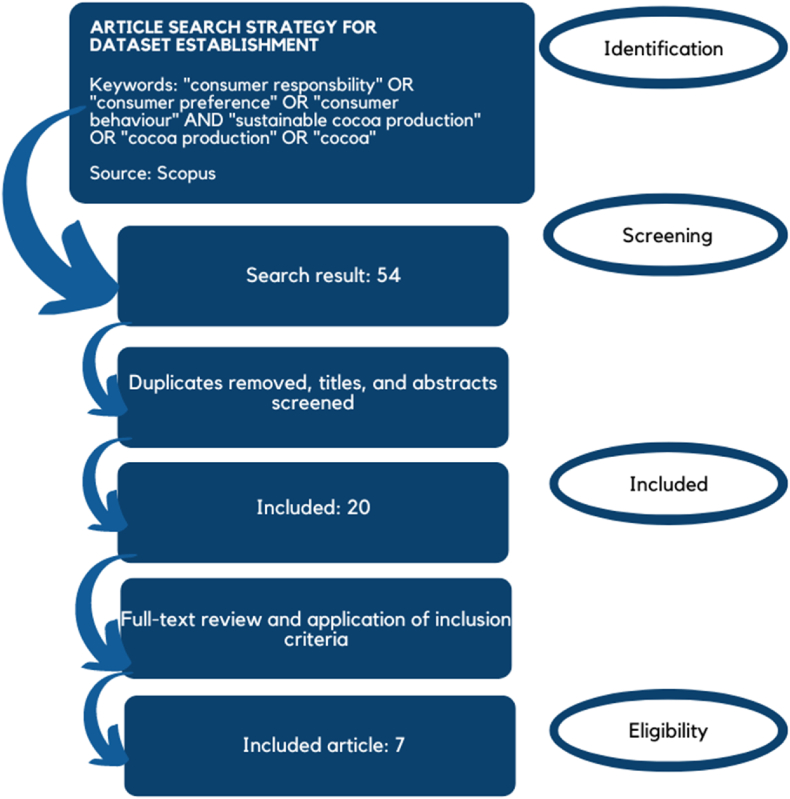
Diagram Flow of the article selection.

The next step involved a detailed review of each article to assess its relevance to consumer responsibility in promoting sustainable cocoa production. After this comprehensive evaluation, 7 final manuscripts were selected as the primary sources for developing this manuscript. This process is illustrated in Fig. 2.

**Fig. 3:**
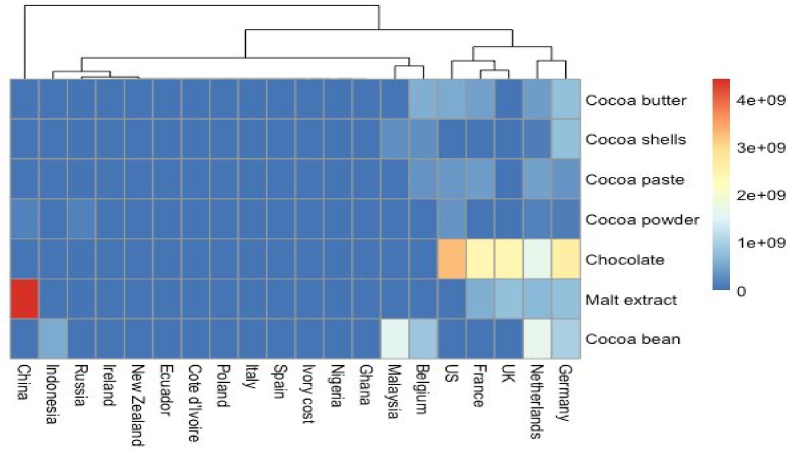
Different types of chocolate come from exporter regions in the world.

## 3 Consumer behavior models in chocolate purchasing

The transformation of cacao beans, derived from the Theobroma cacao L. tree, into chocolate bars entails a comprehensive post-harvest procedure (see Fig. 3). Each stage in this process, encompassing the origin of cocoa, its composition, and the manufacturing methodology employed, has a profound influence on the characteristics of the final chocolate product. These factors bestow upon it distinctive sensory attributes that appeal to consumers. Cocoa-based products enjoy global consumption due to their recognized status as significant sources of polyphenols - molecules that are associated with vital health benefits. In recent times, there has been an observable shift in consumer behavior driven by an increased focus on health and wellness. This trend has led consumers to alter their purchasing habits and seek out products that are associated with beneficial health effects. Within this framework, chocolate with a high cacao content serves as an extraordinary medium for the delivery of bioactive constituents. These include flavonoids, tannins, peptides, fiber, and certain probiotics among others. This elevates chocolate to the status of a health-promoting product due to its nutritional composition. Consequently, the objective of this chapter is to present an updated overview of cacao harvesting and post-harvest management, alongside chocolate production processes. Furthermore, it aims to elucidate how each stage in these processes influences both the sensory attributes and functional quality of the final chocolate product (Galanakis, 2022).

Dietary habits are largely shaped and influenced by an individual's sociocultural milieu (Cicia et al., 2012). Emotions and sensory experiences play a pivotal role in dictating food preferences, leading to the establishment of distinct food choice patterns within specific cultural groups (Codeluppi, 2003). In the realm of psychology, numerous studies have delineated the concept of feelings (physical sensations) as "interpretations of the brain's perceptions of bodily states and alterations," while emotions are described as "processed feelings" (Wachsmuth, 2010; Scherer, 2005).

The dichotomy that exists between emotions and feelings may be compared to the difference between fundamental emotions, also known as basic emotions, and secondary emotions. Primary or basic emotions possess a universal characteristic, being inherently embedded in biological processes, and are also seen in other primate species (Leyens et al., 2001). These emotions comprise a range of affective states, including positive feelings such as pleasure, surprise, and satisfaction, as well as negative ones such as fear, rage, disgust, sorrow, and contempt. The categorization of these fundamental emotions is based on their potential to be interpreted as either a cause or a consequence (Al-thubaity and Alharbi, 2018). Secondary emotions, such as shame, anxiety, loneliness, boredom, and exhaustion, arise as a result of the interplay between main emotions. They undergo evolutionary changes throughout time as a result of human developmental processes and interactions within society (van Kempen et al., 2017; Plutchik, 1994).

Happiness, a subjective emotional state, is characterized by feelings of pleasure, satisfaction, achievement, and general well-being. On the other hand, the negative emotional state of sadness is characterized by feelings of disappointment, loneliness, and a lack of motivation. Therefore, it may be inferred that the presence of a happy emotion results in a matching positive sentiment, whereas the presence of a negative feeling always leads to a subsequent negative sentiment (Cherry and Wilcox, 2020).

The category of chocolate confectionery is a kind of snack food product that often utilizes emotional marketing communication strategies (Liao et al., 2015). The primary objective of these communication methods is to provide pleasurable experiences across the many stages of the shopping process, including the pre-purchase, purchase, and post-purchase phases (Zarantonello and Luomala, 2011). The consumption of chocolate possesses the capacity to elicit psychological changes in individuals, which can manifest as either positive or negative effects, impacting their emotional, cognitive, and sensory responses (Maleki et al., 2020). Emotional eating is characterized as the consumption of food in response to adverse emotions (Steinsbekk et al., 2018; Sultson et al., 2017) and is often associated with emotionally charged foods like chocolate. Such foods are perceived as a refuge from experiencing negative emotions.

In the realm of chocolate, the act of consuming it often elicits a combination of favorable and unfavorable emotional responses. The second category mostly encompasses emotions of shame and guilt that arise from weight increase, sometimes prompted by perceived moral transgressions (Alberts et al., 2012). Regarding consumer behavior in relation to purchasing chocolate, decisions are often driven by feelings or even impulsive reactions. These impulses can be characterized by emotions such as fear, sadness, or anxiety (Willem et al., 2020). Food selections often happen impulsively, driven by quick and spontaneous unconscious processes, particularly when it comes to selecting savory and fatty foods. Nonetheless, modern consumers also make more deliberate informed decisions based on rational thinking incorporating prior knowledge and long-term personal goals into their decision-making process (Kakoschke et al., 2014; Schumacher et al., 2016).

The act of consuming chocolate incites a state of gratification within the individual, thereby establishing a psychological feedback loop that culminates in positive sentiment and may even lead to dependency (Lake, 2015). Factors such as personal traits, mood states, prior purchasing, and consumption experiences significantly influence chocolate consumption patterns and the subsequent emotions elicited by it. Sociodemographic variables along with lifestyle determinants have been identified as influential factors in shaping motivations for selecting emotionally charged foods like chocolate and overall dietary habits (Krieger et al., 2019).

Italy possesses a longstanding tradition in the realm of chocolate production. For regions such as Piedmont and Sicily, the creation of this confectionery item continues to serve as a fundamental component of their national industry. Chocolate production in Piedmont was initiated in 1678, but by the onset of the nineteenth century, chocolatiers were compelled to substitute cocoa with hazelnuts due to the implementation of import tariffs on goods like cocoa by the Napoleonic regime. The globally recognized product, Gianduia (a hazelnut-based chocolate), originated from this region in response to these historical circumstances (Atlante del cibo, 2017; Magli et al., 2016).

Contrary to the chocolate tradition of Piedmont, Sicilian chocolate production primarily distinguishes itself through the product's flavor profiles, rather than its form which is typically a bar or "brick". Modica chocolate, named after the town of Modica in Sicily where it is produced, is characterized by its sugar crystal content and granular texture, a consequence of its unique production process (Lanza et al., 2011). The ongoing diversification in the market with an array of chocolate varieties leads to discernment in quality among chocolate consumers. Viewed from the consumer standpoint, there is a rising trend among Italian consumers towards purchasing premium-priced chocolate produced from single-origin cocoa beans that are often accompanied by Fairtrade and/or organic certifications (MarketWatch, 2020; Pay, 2009).

According to statistics provided by the Centre for the Promotion of Imports from developing nations, the majority of consumers choose to buy chocolate that mostly utilizes cocoa sourced from the Dominican Republic and Ecuador (Ministry of Foreign Affairs, 2022). Along with a taste for dark chocolate produced from certain worldwide cocoa-producing countries, consumers are increasingly interested in chocolates fortified with flavonoids, gluten-free choices, vegan goods, items with lower sugar content, and dark chocolates with extra components.

The selection of food by consumers is greatly influenced by the information shown on product labels. In relation to the labeling of chocolate, European regulations require the chocolate industry to provide essential information, including the percentage of cocoa utilized in the production of chocolate (a minimum of 43%), comprehensive details about the manufacturer (including the name and location of the producer, packager, or seller), a list of ingredients, the expiration date, and the weight of the product (Directive, 2000/36/EC, transposed into Italian law by Legislative Decree 1178/2003). Additional information often seen on product packaging is discretionary and is often indicated when there is an augmentation in the quantity of key components compared to the mandatory recipe.

The comprehension of diverse chocolate kinds among Italian consumers was investigated, with an emphasis on the emotional connotations associated with chocolate choice and consumption. This was further defined in terms of geographical and demographic factors. A consumer survey was undertaken in two geographically separate areas of Italy: Piedmont, which is situated in the northern part of the country, and Sicily, located in the southern region. The aforementioned areas delineate the geographical peripheries of the nation and exhibit distinct populations in terms of socio-demographic characteristics and lifestyle aspects (ISTAT, 2020).

Nonetheless, both populations share a profound and historic tradition of chocolate production, albeit characterized by entirely distinct methods (Gianduia in Piedmont and Modica in Sicily). This could yield intriguing data that mirrors broader consumer predilections (Atlante del cibo, 2017; Lanza et al., 2011). Given the dynamic nature of consumer motivations towards chocolate consumption, it becomes crucial to assess consumer preferences and attitudes towards various chocolate types. This evaluation can facilitate the exploration of emotional marketing strategies aimed at engaging consumers on a personal level, thereby fostering a perception of being recognized, heard, and understood (Merlino et al., 2021).

The European chocolate industry is seeing ongoing growth, mostly fueled by the increasing consumer demand for dark chocolate. Following dark chocolate, hazelnut chocolate constitutes 23% of the consumption, while chocolates with extra ingredients make up 16%. Milk chocolate and white chocolate represent 15% and 4% of the consumption, respectively. The rise in dark chocolate consumption in Italy, similar to patterns seen across Europe, may be ascribed to the increasing inclination of consumers towards healthier choices, as well as their preference for sustainable and premium alternatives, such as certified goods (Del Prete and Samoggia, 2020b; Maleki et al., 2020).

While the enjoyment of chocolate does not necessarily require knowledge about its origin and production conditions, such information is becoming increasingly influential in consumer decision-making processes. Responding to this trend, companies are introducing sustainability labels and providing details about production locations and conditions. For certain products, a compelling narrative becomes the centerpiece of marketing efforts as it can motivate consumers to invest more in the product, potentially even contributing towards its production. The fundamental structure of the chocolate production network has largely remained unchanged since colonial times: countries where cocoa is grown primarily serve as raw material suppliers, while most of the processing occurs in the Global North. This dynamic results in an unequal distribution of labor and value chains that leave only a minor fraction of profits within cocoa-growing countries, along with social and environmental vulnerabilities.

This study explores the preferences and emotional associations of Italian consumers towards various types of chocolate, as well as evaluates which label information influences their purchasing decisions. Notably, a significantly larger proportion of both women and men from Piedmont show a preference for "Gianduia" (a hazelnut-based chocolate), compared to those from Sicily. However, the preferred format of chocolate varies according to gender. Distinct attitudes exhibited before or after consuming chocolate are closely tied to specific product types, with gender and geographical factors playing crucial roles in shaping these attitudes. Both women and men individuals from Piedmont exhibit negative emotions towards chocolate both before and after consuming it. This was demonstrated through the analysis of correlations, which revealed positive associations between feelings of anger before consuming chocolate and subsequent feelings of guilt and sadness in both genders.

During the process of making decisions, a significant proportion of participants, namely over 40%, exhibited a level of consciousness about the cocoa content, nutritional details, and fair-trade certification that were visibly shown on chocolate labels. The aforementioned studies provide empirical evidence that may assist the chocolate business in comprehending consumer sentiments toward chocolate. Additionally, they play a role in raising awareness about the societal consequences of food labeling and laying the groundwork for the selection of a marketing communication plan that effectively utilizes emotional appeals.

Another source showed that in the United States, approximately 22% of consumers opt for chocolate milk (Brodock et al., 2021) making it the most preferred flavor in the country (Boor, 2001; Thompson and Gerard, 2007). However, a significant number of adults perceive commercial chocolate milk as excessively sweet and refrain from purchasing it irrespective of their ethnic backgrounds (Thompson and Gerard, 2007). Paradoxically then, numerous efforts to reduce sugar content in chocolate milk have been centered around maintaining its perceived sweetness and consumer acceptance (Alcaire et al., 2017; Oliveira et al., 2016). This indicates a potential market opportunity for a reduced-sugar variant of chocolate milk that is deliberately less sweet than currently available commercial options. While dark chocolate milk is marketed in several other countries such as the Netherlands (Chocomel®), Denmark (Cocio), and Canada (Natrel), its availability is limited in the US. The dark chocolate milk products provided by small, local creameries in the United States do not exhibit a reduced sugar content compared to conventional varieties. In fact, certain dark chocolate milk offerings, such as those from Anderson Erickson Dairy in Des Moines, IA, and Trickling Springs Creamery in Chambersburg, PA, may even contain higher levels of sugar than standard chocolate milk.

A prevalent issue in the reduction of sugar content in chocolate milk is that the bitterness of the chocolate may become more perceptible when it is not concealed by sucrose (Oliveira et al., 2016). This effect, known as mixture suppression (e.g., Bakke et al., 2018; Lawless, 1979); occurs when a combination of two compounds leads to a lower perceived intensity than what would be expected from each component if they were presented independently. In the context of chocolate milk, it has been shown that a decrease in sugar content is associated with a heightened sense of bitterness, such as the release from mixture suppression. However, this observation does not have a substantial impact on the preference of those between the ages of 18 and 29 (Oliveira et al., 2016).

**Fig. 4:**
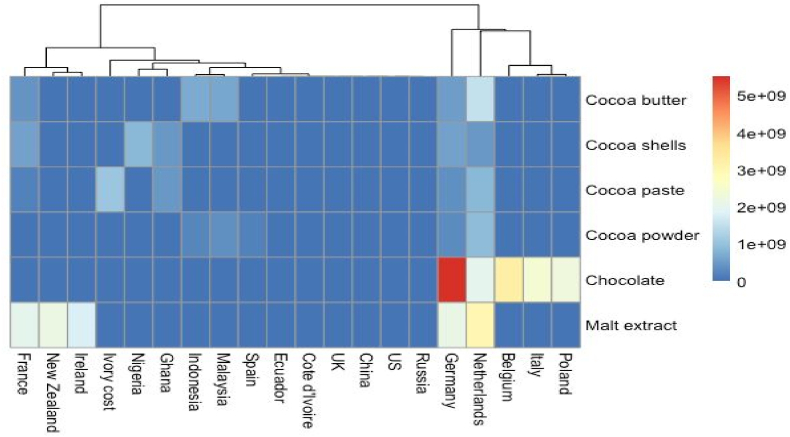
Different types of chocolate come from importer regions in the world.

There exists some empirical data suggesting potential segmentation concerning the bitterness in chocolate milk. Research conducted by Harwood and associates revealed that consumers with a preference for solid dark chocolate over solid milk chocolate also exhibited a higher tolerance for bitterness in liquid chocolate milk (Harwood et al., 2012); It remains uncertain whether these individuals would also favor a more bitter dark chocolate product, but it can be postulated that they might, considering the sale of dark chocolate milk products in other markets (see Fig. 4).

**Fig. 5:**
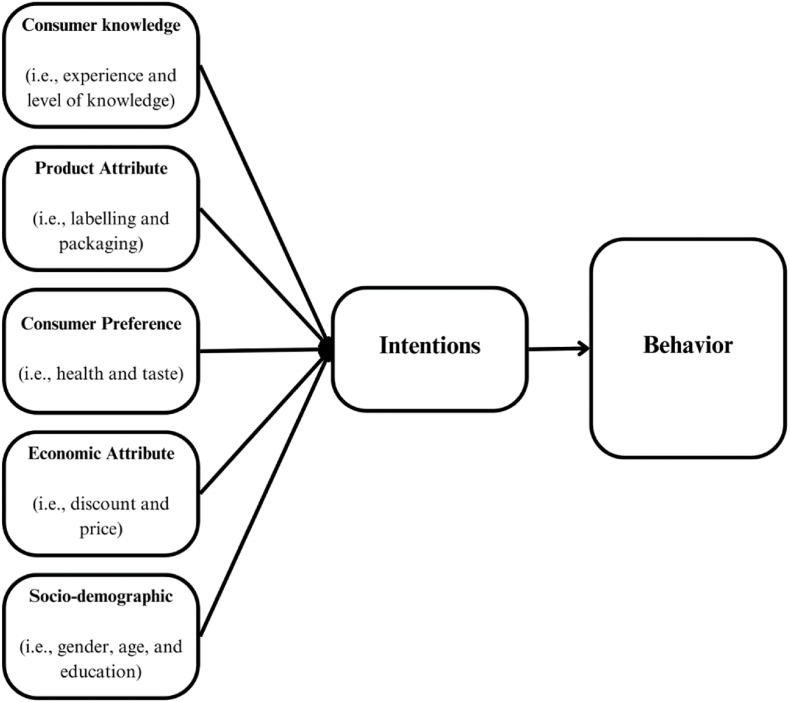
An example concept of the consumer behavior model in chocolate purchasing.

**Fig. 6:**
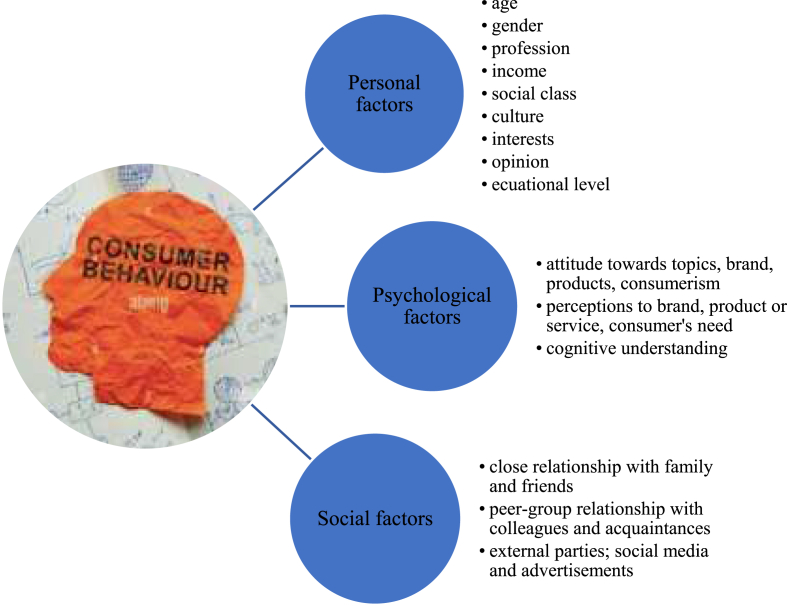
Factors of Consumer behavior.

Moreover, this review uncovered and presented a recommendation model that could be utilized to examine consumer behavior when purchasing chocolate based on the summary of consumer behavior and preferences that could impact decisions when buying chocolate. Subsequently, Fig. 5 presents an illustrative instance of a model pertaining to consumer behavioral model research relating to the purchase behaviors of chocolate. Some of the recommended constructs in the consumer behavior model include consumer knowledge, product attributes, consumer preferences, economic attributes, and socio-demographic factors. The aforementioned constructs have been identified as having aspects that may have an impact on consumer intention and behavior in the context of purchasing chocolate (see Fig. 6).

## 4 The Connection behavior of chocolate consumers to cocoa farmers

### 4.1 Concept of consumer behavior

Numerous research has been conducted on consumer behavior. Consumer behavior determines the buying and spending habits of people which is necessary for marketing tools to identify who the consumers are, who the targeted consumers are, and how and why the consumers behave (Kasza et al., 2022; Madarász et al., 2022; Plasek et al., 2021). Consumer behavior in marketing needs to understand the concept area of demographics, competitors, brands, products and services, consumer habits, and influences. Moreover, consumer behavior is influenced by personal factors, psychological factors, and social factors (Muhammad Tony Nawawi, 2016).

Consumer behavior encloses physical and mental activities that engage the consumer to evaluate, purchase, and use products and services (Cole, 2007). Frequently, consumers are faced among option of product choices before deciding to buy. Therefore, they consider some aspects including brands, quality, color, and material, as well as the surrounding impact, families, groups, salespeople, and others. Two essential inquiries for marketing purposes address consumer behavior such as the way consumers decide on prospective products and aspects that they consider to make decisions (Roy, 2022).

Previous theories mention some consumer behavior models.

#### 4.1.1 Stimulus-response model

Even though many situations influence for process of decision-making, marketing is the most crucial factor of it. The marketing process needs to emphasize the content of the "Black Box" of the consumer and stimulate the transformation into reactions of consumers (Jhonson, 2011). Black box models describe how stimuli, consumer traits, decision-making processes, and consumer reactions all interact. There are two types of stimuli that are often used, namely interpersonal and intrapersonal stimuli. This concept is based on the theory of black box behaviorism, where the emphasis is on the interaction between stimuli and consumer reactions, not on the consumer's internal processes. Marketing stimuli are created and planned by the company, while environmental stimuli are measured based on the economic, political, and cultural situation in a society. The consumer black box encompasses the buyer's attributes and the buyer's decision process, which can influence the buyer's reaction. When marketing concepts and other variables are in the consumer's "black box", they will create specific stimuli (Roy, 2022).

#### 4.1.2 Shet-family decision-making model

Based on Sheth's model of family decision-making, families at the middle-class level, as well as families who are newly married and have strong family relationships and ties tend to practice this collaborative decision-making. Purchasing decisions are easier to make because there is a collaborative system that is mutually agreed upon by family members (Roy, 2022).

#### 4.1.3 Howard – Shets model

The Howard-Shets Model has three levels of learning models, namely expanded problem-solving; limited problem-solving; and routine behavior. When consumers have limited knowledge about the product, they will actively research and compare it with other similar brands. A limited number of problem solutions occur because consumer knowledge and perceptions are only partially formed. Consumers engage in routine response behavior when consumers tend to make purchases from a particular brand based on their knowledge and perception of that brand and its alternatives (Roy, 2022).

#### 4.1.4 Nicosia model

Consumers and potential consumers are the core of this model. Consumers are able to influence business activities which shows an interactive design (Roy, 2022).

#### 4.1.5 Engel Kollat Blackwell model

In this model, consumer purchasing behavior is a combination of the buyer's views and choices. Then the choices before and after purchase are influenced by four variables that are part of this model, namely the decision-making process stage, information input stage, information processing stage, and variables that influence the decision-making process. The processes of problem identification, search, assessment of alternatives, purchase, and consequences are included in the description and decision-making. The Information processing part of the model, which has the first impact on the problem identification stage of the decision-making process, receives information from marketing and non-marketing sources. Consumer reactions, attention, cognition, perception, submission/reception, and retention of incoming or dominated marketer and non-marketing information are all components of consumer information processing (Roy, 2022).

### 4.2 Current Consumer behavior on sustainable perspective

In the context of sustainability, several suitable methods postulate that consumers possess a comprehensive understanding of the environmental attributes of a product prior to engaging in purchase behavior. Furthermore, as a result of the intricate nature of these environmental characteristics, buyers may possess an incomplete comprehension prior to making a purchase. The presence of ambiguity has the potential to result in post-purchase unhappiness, hence impacting consumer utility (Bell, 1985). Moreover, the discrepancy between anticipated value and actual outcome can significantly influence consumer utility (Inman et al., 1997). For eco-label regulations, when manufacturers self-report product greenness using individual labels, the abundance of different labels can lead to consumer confusion, making label comprehension more difficult (Brécard, 2014).

The potential for diminished value in the adoption of labels may arise due to confusion (Harbaugh et al., 2011). Furthermore, the presence of competition across labels may lead to limited environmental advantages (Fischer and Lyon, 2014). In the present setting, in order for consumers to have a comprehensive understanding of the environmental sustainability of a product prior to making a purchase, a significant amount of research and inquiry is necessary. In this particular scenario, the perception of eco-labels by consumers might be subject to the effect of their own level of environmental consciousness, hence resulting in a divergence in the perceived worth of an eco-label among different persons. An eco-label is established by an autonomous entity such as a non-governmental organization (NGO), to enable both the NGO and the manufacturers to use the mark to educate consumers, hence fostering environmentally conscious behavior.

As previously said, eco-labels established by non-governmental organizations (NGOs) provide consumers with a more convenient means of comprehending the environmental attributes of products. This is achieved by the provision of information on NGO websites, therefore facilitating a straightforward and economical process for consumers to acquire knowledge on the subject matter. In brief, there is a limited body of research investigating the unique consumer behaviors associated with company self-labels and NGO eco-labels, and the subsequent impact on sustainable production under varying regulatory frameworks.

While existing work has examined consumer purchase behavior in relation to eco-labeled items, there is a dearth of mathematical models that specifically demonstrate the usefulness of consumer purchasing behaviors in the context of producer self-labels and NGO ecolabels. Significantly, when consumers are required to conduct a thorough examination of a product before making a purchase, this undertaking involves incurring a research cost that is not taken into account when engaging in a direct purchase (Kavaliauske et al., 2014). Insufficient attention has been given to the consequences pertaining to consumer environmental consciousness and sustainable consumption strategies, which are no longer viable in the current intricate context of emission reduction and supply chain frameworks. In terms of substance, the prevailing body of literature mostly focuses on the examination of traditional environmental laws, such as environmental taxes, green subsidies, carbon quotas, and third-party label certification (Gao and Wei, 2023).

### 4.3 The relation between consumer behavior with cocoa farmers

Cocoa farmers are expected to produce high-quality cocoa beans to meet consumer demand. For the global market, cocoa dryness standards are one of the determining factors for the quality of cocoa beans recognized by global consumers, especially industry players. In an effort to fulfill consumer desires, several regulations have been tried by both the government and NGOs to increase the added value of cocoa. One of them is implementing a cocoa certification program. The certification program is the standard for consumer needs for dry cocoa beans produced by cocoa farmers. This certification program is implemented in the form of a partnership between the certification body and cocoa farmers in a sustainable cocoa program. In this case, the certification body collaborates with the industry which provides market certainty to farmers with higher prices (Iddrisu et al., 2020).

The sustainability program through cocoa certification is a form of creating a relationship between chocolate consumers and cocoa farmers. Consumers need good quality cocoa products through partnership programs by both the government and NGOs in the form of collaborative partnerships to produce quality cocoa beans by farmers. In order to produce premium chocolate for consumers, of course, it starts with producing quality cocoa beans by cocoa farmers. In the agribusiness concept, to achieve the downstream sub-sector, in this case, good marketing, it is very important to improve the downstream system, in this case providing quality raw materials. Because the raw materials are produced by cocoa farmers, with all the limitations they have, they require a supporting sub-system, either government or NGO, in order to produce raw materials for cocoa beans that suit the needs of chocolate consumers (Herman, 2020).

Therefore, implementing the concept of sustainability through cocoa certification is an effort made to connect the needs of chocolate consumers and cocoa farmers. One of the certification bodies that applies the concept of sustainability to farmers is UTZ-certified. This UTZ-certified certification institution projects its programs to strengthen local products and global value chains. As a world certification institution, UTZ-certified sets standards for the production and purchase of responsible agricultural commodities. (Herman, 2020; Iddrisu et al., 2020).

UTZ-certified guarantees professional, social, and environmental quality in the production practices expected by brand owners (buyers) and consumers. By implementing a professional work system for cocoa smallholder farmers, the UTZ-certified program starts from on-farm, which is the selection of locations for cocoa cultivation, as well as the selection of seeds, maintenance, and fertilization. In the certification program, the sustainability program to be achieved includes three aspects, that are economic, social, and environmental. This certification program will benefit both partners who collaborate. For smallholder farmers, the benefits include an easy-to-get better market, premium cocoa price, and training programs to increase smallholder farmers' capacity. This sustainability program had a good performance in the economy and environment but it is still bad in social aspects (Karim et al., 2020).

In catering to the empathetic sensibilities of global consumers, particularly those in Switzerland, the country presents a promising market for fair-trade and organic cocoa sourced from farmers. This is largely due to the strong ethical inclination among Swiss consumers towards purchasing such products. In 2020, Swiss per capita expenditure on organic products (including chocolate) was estimated at €418, marking it as the highest globally. Organic retail sales in Switzerland totaled €3.6 billion within the same year. With an organic market share of 11% in 2020, Switzerland ranks third worldwide in this regard. The Research Institute of Organic Agriculture reports that between 2016 and 2020, Swiss organic retail sales experienced an average annual growth rate of 12% (Statista, 2023).

## 5 The empathy behavior of chocolate consumers to cocoa farmers

### 5.1 Relevant concept of empathy behavior

Empathetic behavior plays a significant role in shaping interpersonal relationships, leading to either positive or negative outcomes. From both biological and psychological perspectives, empathy is considered crucial for human survival and is often associated with success in life. Empathy is a comprehensive concept encompassing the cognitive and emotional responses of an individual to the observed experiences of another person. The presence of empathy enhances the probability of offering help and demonstrating compassion towards others. It serves as a fundamental component of morality for individuals. Moreover, it is an essential element in successful relationships as it aids in understanding others' viewpoints, needs, and intentions.

The evolution of various social competencies throughout an individual's lifespan is contingent upon empathic functioning (Trivedi-Bateman and Crook, 2022). Historically, empathy has been conceptualized in a multitude of ways, with as many as 43 distinct definitions found in scholarly literature. However, it is generally recognized as a multidimensional psychological construct that enables individuals to both identify (cognitive) and react (affective) to the emotional states of others (Cuff et al., 2016). More recently, some researchers have proposed an alternative three-dimensional model of empathy with enhanced psychometric attributes. This model includes cognitive empathy, emotional contagion, and emotional disengagement (Herrera-López et al., 2017).

Variations in empathic responses among individuals are shaped by a blend of both state (contextual) and trait (dispositional) factors (Cuff et al., 2016). Multiple methodologies are employed to assess empathy, including several standardized scales such as the Interpersonal Reactivity Index (Keaton, 2017) and the Basic Empathy Scale (Jolliffe and Farrington, 2021). Empathy is also commonly evaluated using hypothetical situations where individuals' behavioral outcomes are analyzed as indicators of their empathic capacity (Hein et al., 2018).

### 5.2 Consumer empathy behavior

Consumer empathy is increasingly becoming a focal point for both brands and marketing insights professionals. The endeavor to comprehend and implement it within their communication strategies is widespread. The phenomenon of empathy, which pertains to the capacity to comprehend and appreciate the emotional state of another individual, is widely seen (Hossain and Rahman, 2022a). The concept of empathy presents a challenge since it seems to contradict the prevailing notion that emotions primarily serve to further our own goals and aspirations. It has been argued that empathic emotions are more aptly applied to the circumstances of others rather than our own (Abramson et al., 2020). Nevertheless, it is worth noting that we may sense emotions toward others even when our own goals are not at stake. This phenomenon is comparable to the experience of emotions that arise when we see a remarkable piece of artwork or when we take a leisurely walk outside on a bright day (Wondra and Ellsworth, 2015).While trendy topics often lose momentum after a certain period, fundamental human principles should not be relegated to the realm of forgotten buzzwords. Marketing initiatives lacking in empathetic understanding are destined for failure. However, there is encouraging news - empathy can be cultivated by brands and the individuals behind them. Modern consumers value emotional, experiential interactions. They frequently do not simply purchase 'products' or 'brands.' Instead, their purchasing decisions are guided—now more than ever—by their ethical beliefs. Brands that are committed to authentically understanding their consumers' aspirations and values stand a better chance of securing consumer loyalty, trust, and financial support. The complexity of consumer empathy escalates within our diverse cultural environment. Our society consists of emotionally driven individuals who fuel our economy. These consumers anticipate—and often insist—that brands take a stance on public figures or express their position on controversial issues(Bonilla, 2015).

Emotions may be seen as psychophysiological reactions that occur in response to environmental stimuli (Bhandari et al., 2019). The cognitive processes, attention, behavior, and choices of individuals may be altered by these responses, therefore facilitating coping, adaptability, and overall well-being (Zou et al., 2021). Discrete emotions, like happiness, anxiety, rage, and sadness, are characterized by their distinct and concentrated nature. However, it is important to note that an individual's total affective state encompasses a spectrum of both positive and negative emotions (Septianto and Chiew, 2018). An emotional experience may be defined as a conscious and subjective phenomenon that is primarily characterized by psychological manifestations, mental states, and physiological reactions (Turner, 2009). Based on the prevailing body of research, emotion may be characterized as a positive or negative subjective experience that is associated with a certain set of physiological responses. The examination of consumers' emotional experiences in relation to their related behavior has been conducted (Zou et al., 2021). The impact of associated emotions, particularly those associated with consumers' motivation or decision processes, has been empirically shown to exert an effect on many aspects of the overall experience (Chuang & Lin, 2007). The emotional experiences that consumers encounter have the potential to impact both their levels of satisfaction and their subsequent behavioral intentions (Zou et al., 2021).

Environmental psychology postulates that factors such as physical environments, architectural design, and service interactions significantly influence the consumer experience (Bitner, 1992). Studies in environmental psychology that focus on consumer experiences provide evidence for this perspective (Donovan and & Rossiter, 1982). Mehrabian and Russell (1974) developed the Stimulus-Organism-Response (SOR) paradigm acknowledging the advantage of applying environmental psychology to consumer evaluations. This model has been utilized extensively within marketing and consumer behavior disciplines (Jang and Namkung, 2009; Kimiagari et al., 2021; Hossain and Rahman, 2022b).

### 5.3 Sustainability program as a social empathy behavior for cocoa farmer

Therefore, it can be seen that empathy is a result of emotional experiences. Empathy is a broad and encompassing notion that pertains to the cognitive and emotional reactions of a prospective consumer toward the perceived experiences of others. The concept of empathy encompasses a diverse array of phenomena, including but not limited to demonstrating concern for others and a desire to assist them, experiencing emotions that parallel those of another individual, discerning the thoughts or emotions of another person, and dissolving the boundaries between oneself and others (Kamas and Preston, 2021). Empathy is a social ability to understand relationships which has three skill types; expressive skills, sensivity, and control communication. One of the empathy behaviors that comes from external support to lead cocoa farmers is how to help them produce high-quality cocoa beans to meet market demand. Without any help, cocoa farmers potentially found a lot of obstacles to present good quality standards of cocoa beans. Cocoa farmer is the main producer which needs support from stakeholders. It is such an empathy program from the social actors as a good approach for cocoa stakeholders to meet market demand in the cocoa sector.

Fairtrade is a collaborative trading relationship that emphasizes dialogue, transparency, and respect with the aim of achieving increased equity in global commerce. It fosters sustainable development by providing improved trading conditions and safeguarding the rights of disadvantaged producers and laborers, particularly those in the Southern Hemisphere. Furthermore, organizations certified as fair trade must adhere to a set of criteria pertaining to social, economic, and environmental developments. Additionally, these organizations are required to maintain certain standards within their operational conditions.

A defining feature of fair trade cocoa is that it provides producer organizations with a higher price for their cocoa beans. This elevated price is critical for these organizations to have the financial capacity to meet the stipulated requirements and cover certification costs. The fair trade price is determined based on global market prices, in addition to fair trade premiums. The standard quality cocoa commands a fair trade premium of US$ 150 per tonne. Including the premium, the minimum price set for standard quality fair trade cocoa stands at US$ 1750 per tonne. Additional advantages for cocoa farmers in certified producer organizations include enhanced capacity building and improved market access. However, currently, cocoa bearing the fair trade label constitutes a relatively small portion of the overall cocoa market, approximately 0.5% (ICCO, 2021).

Therefore, one of the institutions supported local cocoa farmers through the sustainability concept. In this program, the UTZ-certified institutions collaborated with smallholder farmers to certify cocoa beans in the Indonesian case study. On a micro level, cocoa in Indonesia is a livelihood source for farmers in rural areas which increases the family's income (Herman, 2020). UTZ-certified not only exists in Indonesia but also in Ghana as a cocoa producer as well (Ingram et al., 2018). UTZ has been certifying cocoa beans of smallholder farmers in Indonesia since 2011 till now. The UTZ-certified provides certification standards for smallholder farmers such as 1) occupational safety and health for smallholder farmers, 2) The use of pesticides that are good and safe for cocoa farmers, 3) quality control and post-harvest of cocoa plants, 4) good agricultural practices of cacao, 5) control of pests and diseases of cocoa plants which is one of interconnected problems in cocoa industry such like the repercussions of disease and pest infestation (Matissek et al., 2012).

The objectives of these programs are to ensure the preservation and enhancement of soil fertility and structure, safeguard plant health, and effectively manage agrochemical storage. The measures involved encompass various stages such as harvesting and post-harvesting activities, guidelines for pesticide usage, a compilation of recommended plant protection products along with their application instructions, the implementation of the Integrated Management/Integrated Pest Management (ICM/IPM) approach, provisions for occupational health and safety of workers/farmers, protection of labor rights, and conservation and preservation of the environment. UTZ-certified engages in partnerships with smallholder farmers, as well as Swisscontact, a Swiss non-governmental organization specializing in the cocoa industry. The objective of these collaborations is to enhance the competitiveness of farmers within the cocoa value chain, hence augmenting on-farm cocoa bean production (Schaad and Fromm, 2018). These stakeholders also cooperate to increase market access for smallholder farmers. Thus, productivity and promotion can guarantee high quality and quantity to meet market needs, as long as to secure the e livelihoods of smallholder farmers (Matissek, Reinecke and Hagen, 2012).

The sustainability program is elaborated specifically into 20 activities implemented by UTZ Certified as a certification institution for smallholder farmers. The programs are as follows.

Certification programs which is leaded by UTZ Certified are 1) a good way to prepare cocoa farm, 2) provision of cocoa seedlings and location, 3) raining/information related to the maintenance of cocoa plants, 4) training/information related to proper fertilization, 5) information on cocoa pest and disease control standards, 6) information about the procedures for harvesting and post-harvesting according to certified standards, 7) preparation of cocoa beans warehouse and storage, 8) information about proper harvesting, 9) the availability of cocoa beans drying and the equipment, 10) the availability of cocoa bean fermentation, 11) utilization of cocoa bean dryer technology, 12) cocoa bean sorting is available, 13) training on the use of tools of cocoa farming, 14) safety standards and use of pesticide mixtures, 15) provision of locations and disposal sites for excess pesticides, 16) introduction and training in pest control methods and methods, 17) provision of good sanitation, 18) training how to control cocoa diseases, 19) food consumption that are recommended to smallholder farmers, and 20) clothing standards used in cocoa farming.

Overall, based on 20 activities of the partnership program between UTZ-certified and smallholder farmers showed high performance with an average value of 78.03%. It relates to another research which indicated that farmers are satisfied with UTZ certification, it represents an opportunity to produce better quality cocoa beans (Schaad and Fromm, 2018). Cocoa bean quality has been increasing generally, due to the training of farmers, increasing use of drying equipment, market access, and regulatory standards (Ingram et al., 2018). These programs above are part of Good Agricultural Practice (GAP) in which it is important to apply by smallholder farmers to increase their capability as the main producers of cocoa beans in Indonesia.

The same way is implemented by UTZ-certified. The UTZ also implemented training of smallholder farmers to maintain cocoa plants, to proper fertilization, and to use the tools of cocoa farming, the availability of cocoa beans drying and the equipment as well as beans fermentation and also utilization of cocoa bean dryer and sorter technology. Moreover, the best planting material with appropriate fertilizer supply, comprehensive farm management practices, and sufficient (Cryer et al., United Kingdom). This model collaboration through 20 activities of the program which was applied by UTZ-certified will maintain smallholder farmers as cocoa producers to produce high-quality cocoa beans in Indonesia. Actually, the collaboration not only had a good impact on smallholder farmers but also on UTZ-certified as a certification institution. In economic aspects, quality product guarantees and increased income, and in social aspects; cooperation is expected to be sustainable (Suriati et al., 2015). Whereas, certification institutions had strong access to the straight market or cocoa industry which guaranteed to buy the cocoa beans at an ideal price.

**Fig. 7:**
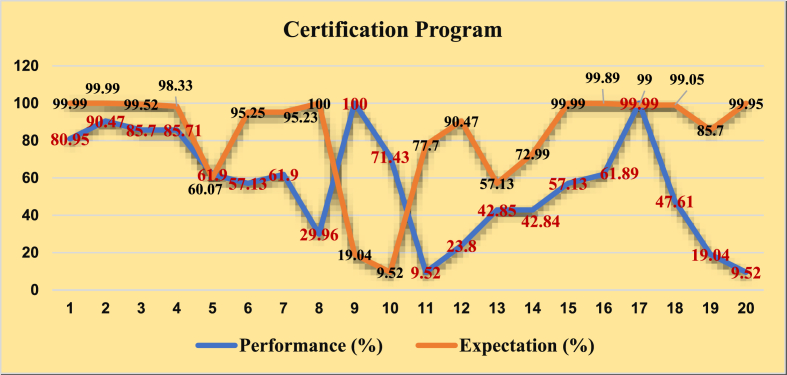
Performance of UTZ certified and expectation of smallholder farmers.

However, In the Indonesian case, some activities were implemented, especially, the utilization of cocoa bean dryer technology, clothing standards used in cocoa farming, information about proper harvesting, and food consumption that is recommended to smallholder farmers, and cocoa bean sorting is available which the performance is under 30%. Only two activities, in this case, the availability of cocoa bean fermentation not really important to implement based on smallholder farmers' perception with a percentage of 9.52 %, and the availability of cocoa beans drying and the tools with a percentage of 19.04 % while the other activities average reached above 50%. It means smallholder farmers have high expectations to continue the certification programs, as well as improve their performance. Another perception in Ghana is that to increase the performance of cocoa, it needs to engage the government, donor agencies, and civil society to involve young cocoa farmers with the aims of increasing national production as well as securing the future of the Ghanaian cocoa industry (Amon-Armah et al., 2017) (see Fig. 7).

## 6 Consumer sensitivity to the environment in cocoa production

**Fig. 8:**
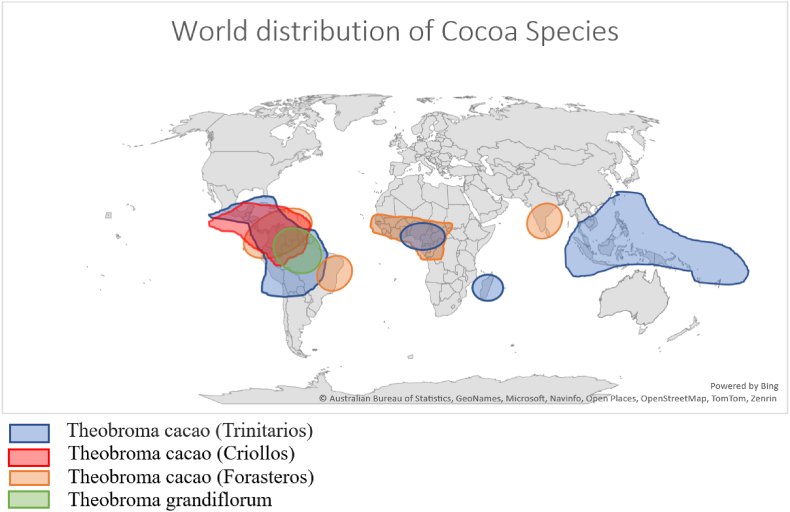
Approximate location of T. cacao Species.

The cocoa cultivation phase is the start of a multi-phased process that is known as the chocolate production process. The chocolate producing process reveals the existence of four distinct species of cocoa that are distributed throughout various regions worldwide (Alves et al., 2007; Bekele et al., 2020; Elwers et al., 2009; Lachenaud and Motamayor 2017; Lim 2012). Subsequently, Fig. 8 will provide a comprehensive depiction of the global distribution and ancestral origins of several cocoa species.

Moreover, the harvesting, extraction, fermentation, drying, and packing of the cocoa beans are the next steps. The dried and fermented cocoa is then gathered and shipped to the factory that makes chocolate (Hosseinzadeh-Bandbafha and Kiehbadroudinezhad, 2022). The cocoa production process is linked to several environmental issues such as the emissions of greenhouse gas, deforestation, water pollution, waste generation, and soil erosion (Konstantas et al., 2018; Nieburg, 2015). As a result, consumers are more interested in purchasing more sustainable chocolate products (Del Prete and Samoggia, 2020a,b). Throughout the chocolate production process, environmental issues are crucial, and businesses are realizing that responsible cocoa production not only constitutes an ethical issue but also a financial one. A more sustainable approach can help address these environmental challenges in order to render the industry long-term viability (Drew and Boal, 2019).

In order to promote environmentally-conscious cocoa production, certifications, and labeling were reported to play a very important role (Silva et al., 2017). Sustainable agriculture and environmental preservation have been connected to certifications like organic and fair trade (Haynes et al., 2012). Also, by tracing the cocoa supply chain, a problem like deforestation is addressed and cocoa farmers' livelihoods are maintained (Renier et al., 2023).

### 6.1 The cocoa production process

Principally, there are 5 different steps involved in the production of chocolate: mixing, refining, conching, tempering, and molding. Every step in the chocolate manufacturing process is significant. The correct manufacturing process parameters must be chosen, together with high-quality raw ingredients, to produce chocolate of the intended standard. By using a three or five-cylinder system in the refining process the correct particle size is achieved and, as a result, the ideal smooth texture in the finished product. The physical properties and sensory characteristics of the finished product are also impacted by this procedure. Conching is done by combining the chocolate ingredients at high temperatures, usually >40 °C. The smoothness, taste, and consistency of chocolate are influenced by conching duration and temperature. During the crystallization process, lipids create a solid structure that has an impact on a number of the significant functional and physical properties of chocolate. Other quality attributes including color, rigidity, and storage life are impacted by tempering (Gursoy and Heperkan, 2020). Making a paste out of ingredients like sugar, cocoa liquor, and cocoa butter is the initial stage of making chocolate. Using two- and five-cylinder refiners, this combination, which is created with an oil concentration of 8–24%, is refined to a particle size of under 30 μm. The final product's physical properties and sensory qualities are substantially impacted by the size of the particles (Gursoy and Heperkan, 2020). Chocolate gives the mouth a gritty feel if the particle size is greater than 30 μm, and a putty texture if the size of the particles is less than 30 μm (Rousseau, 2016). Conching is the next technique applied to the preceding mixture to improve the finalized product's flavor, texture, and consistency (Gursoy and Heperkan, 2020). Conche is a mixer that improves the end product's taste and makes sure the chocolate bulk turns into liquid. The cocoa bulk's sour taste and wetness are reduced by blending. Conching time and temperature are crucial and have a significant impact on the finished product's softness, flavor, and other qualitative attributes and also vary according to the type of chocolate (Rousseau, 2016). Three steps of conching must be completed to produce a high-quality bar of chocolate: Dry conching, pasty phase, and liquefying. The chocolate is powdered and has a high water content during the dry conching step. The fluidity of the substance is badly impacted by too much wetness. This step is crucial to attaining the proper fluidity because, during the dry conching stage, the oil does not entirely coat the face of the chocolate powder (Gursoy and Heperkan, 2020). At this point, the powder is quickly taken at high temperatures and blended to create a chocolate with less wetness. The cocoa butter in the chocolate grains starts to liquefy when it is heated, and the granules join together to create a mushy texture. Conching is now in its liquefying phase. Lastly, during the wet conching stage, oil and emulsifiers are included in the chocolate to give it the required movement qualities for the subsequent refining procedures (Gursoy and Heperkan, 2020). Tempering impacts quality traits including color, solidity, and storage life, and is crucial for chocolate to be in a properly assorted structure. Tempering may be done using four fundamental processes. The process begins with full melting at 50 °C, then cooled to the crystallization point at 32 °C, crystallization occurs at 27 °C, and all fragile crystals are transformed between 29 and 31 °C.

Due to the differing influence of milk fat on the crystallization process in milk chocolate, the tempering process is different between dark chocolate and milk chocolate (Garti and Aserin, 2012) (see Table 2). The main components of chocolate are milk powder, sugar, cocoa liquor, cocoa butter, and cocoa powder. But, the variety in chocolate comes from adding other components like nuts, dried fruits, or grains. The three primary types of packaged chocolate are dark, milk, and white; each has a unique amount of cocoa powder, milk powder, and cocoa butter. The components' proportions in white, milk, and dark chocolate are given in Table 3.

**Table 2:** Chocolate manufacturing processes and their impacts on the environment.

Processing steps	Main features	Environmental Impacts	References
Harvesting and breaking of the pods	The harvest is executed using different long-handle tools (machete, pruning shears, etc.) to get the pods from cocoa trees. It takes place based on the color of the pod of cocoa when they are at maturity. This happens normally after 4 years on average. Exactly 5 days after harvesting the workers slice open cocoa pods and collect white pulp and seeds. One pod can contain about 20–50 beans as per its variety.	Deforestation, Soil degradation, Water pollution, Greenhouse gas emissions, Energy use, Biodiversity loss	(Cassiday, 2012);
			(Kokou, 2014);
			(Hii et al., 2009);
			(Gockowski and Sonwa, 2011),
			(World Wildlife, 2017; Aliouche, 2023; Fountain and Hütz-Adams, 2015);
			(Hoare et al., 2017; NWF International, 2022);
			(Amponsah-Doku et al., 2022);
			(Nieburg, 2015; Bianchi et al., 2021).
Fermentation and Drying	Fermentation is necessary for the removal of the seeds' ability to bud and produce aroma and flavor. At this step, the quality of the cocoa powder is determined. After fermentation, the beans are dried either naturally using solar drying in the sunlight or artificial dries that use the heat produced by a furnace. This step is done to protect the beans from degradation by bacteria.	Greenhouse gas emissions, water use, air pollution	(Adeyeye et al., 2010);
			(Afoakwa et al., 2008);
			(Suazo et al., 2014);
			(Krysiak, 2006);
			(Kokou, 2014; Delgado-Ospina et al., 2020);
			De Vuyst and Weckx (2016)
Packaging and shipment	After drying, the beans are loaded into bags for storage in warehouses before exporting to diverse locations around the world.	Greenhouse gas emissions, Energy use	(Beg et al., 2017),
			(Kokou, 2014);
			Bianchi et al. (2021)
			(Mowbray, 2022);
			(The Climate Collaborative, n.d.; Feber et al., 2021; foodprint, 2018);
			(Leblanc, 2023; Boakye-Yiadom et al., 2021);
			Konstantas et al. (2019)
Roasting	Roasting is carried out once the dried and fermented beans arrive at the processing area. This step helps further develop the flavor of cocoa. The cocoa beans treatment is carried out at a relatively high temperature of 95–145 C. This process helps loosen the shells, removing the moisture from the beans and developing the final color and flavor of the beans.	Greenhouse gas emissions, energy use, air pollution	(Oliviero et al., 2009; Krysiak et al., 2013; Krysiak, 2006);
			(Ioannone et al., 2015; Afoakwa, 2014; Konstantas et al., 2018);
			(Bianchi et al., 2021; World Wildlife, 2017);
			(Nieburg, 2015; La Mantia et al., 2023)
Kibbling and winnowing	In these steps, the shell and the nibs are separated. Kibbling refers to the process of smashing roasted beans. After kibbling, the nib can be separated through winnowing which uses the difference in density to isolate the shell from the nib.		(Ioannone et al., 2015; Anang et al., 2013; Afoakwa, 2014; World Wildlife, 2017; Konstantas et al., 2018);
			(Vásquez et al., 2019);
			Nieburg (2015)
Grinding	At this stage, the nibs are ground into a paste. The cocoa liquor can also be produced at this stage through the melting of the cocoa butter at temperatures above 34 °C in the nib. Then all the chocolate ingredients (sugar, cocoa liquor, etc.) are mixed to obtain a proper mixture and texture.		(Samanta et al., 2022; Afoakwa, 2014; Konstantas et al., 2018);
			(Aliouche, 2023);
			(Nieburg, 2018; Bianchi et al., 2021)
Conching and Tempering	In this step, the last mixing and heating are carried out. During conching, the appropriate viscosity is attained, and all unwanted flavor and moisture are removed. After this step, through heating and cooling tempering is done to obtain crystals of cocoa butter and then to form a stable crystalline network.	Energy use, greenhouse emissions, waste generation	(Afoakwa et al., 2008);
			(Aprotosoaie et al., 2016);
			(Afoakwa, 2014);
			(Di Mattia et al., 2014);
			(Serena, 2021; Barišić et al., 2019);
			(Bianchi et al., 2021; manufacturing.net, 2016);
			Helenthehare (2014)

**Table 3:** Ingredient percentages for dark, milk, and white chocolate are based on assumptions.

Ingredient	Cocoa butter	Cocoa liquor	Cocoa powder	Milk powder	Sugar	References
Dark	28%	42%	16%	0%	14%	Bianchi et al. (2021)
	20.2%	50.1%	16.0%	–	13.7%	Recanati et al. (2018)
	8.5%	47.5%	–	–	43.5	Boakye-Yiadom et al. (2021)
	–	40%	–	–	45%	YCW (2013)
	–	60%	–	–	39.5%	Stuart (2014)
	–	65–99%	–	–	1–40%	Willow (2022)
	4%	66%	–	–	29%	Latner (2016)
White	35%	0%	0%	20%	45%	Bianchi et al. (2021)
	30–45%	0%	–	25–40%	25–55%	Willow (2022)
Milk	15%	25%	0%	20%	40%	Bianchi et al. (2021)
	–	35–55%	–	20%	0–25%	Willow (2022)
	18%	42%	–	14%	25%	Latner (2016)
	17%	8%	–	24.5%	45%	Konstantas et al. (2018)
	20%	18%	–	30%	30%	Boakye-Yiadom et al. (2021)
	–	10%	–	25%	45%	YCW (2013)
	19%	12%	–	20%	48.6%	Stuart (2014)

**Table 4:** Features of CSR strategies that major chocolate producers throughout the world have adopted.

Company	Invented	Areas of corporate business	References
	names of CSR strategy	responsibility (acc. to ISO 26000)	
		Contributions to society and development of the local community	
Mondelēz International	Cocoa Life	*Supporting communities' ability to decide how they want to grow. *Support for creating a community action plan Improving the position of women in society*Supporting education *The next generation will find cocoa production more appealing.	Mondelez International (2012)
Nestlé	The Nestlé Cocoa Plan	*supporting children's education * improving the status of women in society	Nestlé (2019)
Mars Wrigley Confectionery	Cocoa for generations	* improving the position of women in the community	Mars Wrigley Confectionery (2019)
Ferrero Group	Glocal Care Ferrero	*training for farmers	Ferrero Group (2017)
		* preservation of health, encouragement of education, and social advancement	
Lindt & Sprüngli AG	Lindt & Sprüngli Farming Program	* larger impact on enhancing standards of living	Lindt & Sprüngli, 2019
		* Infrastructural development for local households	
		* encouraging education	
		* supporting projects for equality	
Hershey Co	Shared Goodness	*Local community involvement in community projects	The Hershey Company (2019)
		* Greater educational availability	
		* Better living standards	
Meiji Co Ltd	Meiji Group CSR 2026	*Enhancing the status of women in the organization and providing assistance to those with disabilities	Meiji Holdings (2019)

**Fig. 9:**
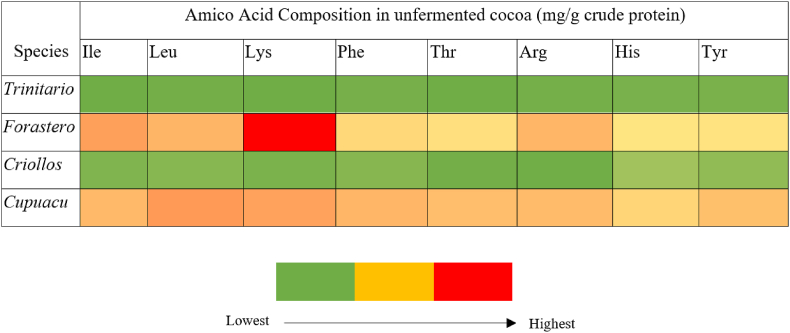
Specific cacao species' amino acid concentration in unfermented cocoa mg/g crude protein (Adeyeye et al., 2010; Korcari et al., 2023; Niemenak et al., 2020; Rogez et al., 2004).

Understanding the amino acid composition of cocoa has significant importance within the chocolate production process (see Table 4). This is quite advantageous as it will significantly impact the composition of the remaining chemicals and beneficial constituents of cacao for the human body. The presence of production flaws, such as inadequate cacao heating procedures, might result in adverse consequences for the remaining crucial components found in cacao. Subsequently, Fig. 9 presents a concise overview of the amino acid content in unfermented cocoa, expressed in milligrams per gram of crude protein. This information serves as a point of reference for the improvement of cocoa processing techniques.

### 6.2 Environmental impacts

The environmental consequences associated with cocoa production are evident at several stages of the value chain, including the cultivation of agricultural inputs and the industrial processing of cocoa beans to yield cocoa flour. Certain consequences may manifest promptly, shown by the poisoning of water sources or the depletion of biodiversity as a result of the use of chemical pesticides (Takyi and Amponsah, 2020). There are other ramifications that may not be immediately apparent, such as the production of greenhouse gases during the transportation of raw materials to the manufacturing facility, which is facilitated by the use of fossil fuels. According to reports, deforestation emerges as a prominent local environmental repercussion of the chocolate industry, especially when cocoa farmers choose to replace tropical forests with new tree plantations instead of using land reuse strategies. According to estimates, the cultivation of cocoa is responsible for around 70% of illegal deforestation in Cote d'Ivoire (World Wildlife Magazine, 2017). Apart from this, the loss of soil fertility and quality is a localized consequence where inappropriate agricultural farming techniques are used (Tondoh et al., 2015). The environmental implications of cocoa production are inextricably linked to other types of exploitation. The most serious is the use of child labor in the cultivation, harvest, and transportation of cocoa beans. Slave labor is also present in the sector, as individuals work on farms but are not paid (Tony's Chocoloney, 2021). It should be noted that regardless of these types of damage, the cacao sector has reasons to be optimistic. The significance of tropical forests in reducing emissions, for example, is receiving prominence in the climate debate, which has led to actions to safeguard tropical forests (Tompkins, 2021). One potential remedy to deforestation is to engage in agricultural practices that improve the efficiency of current farms, lowering the motivation to destroy new forests (Ingram et al., 2018). Furthermore, institutions can fund the changeover phase, thus, rendering forest restoration financially feasible for farmers (Nieburg, 2015). In the following sections, a more thorough investigation of the agricultural process is carried out since cocoa provenance has been influencing consumer choice more and more in recent years (Torres-Moreno et al., 2012). According to the Product Category Rules (PCR), there are indicators for resource consumption and environmental impacts which are: the water deprivation potential (WDP), the abiotic depletion potential (ADP, ff) for fossil resources, the abiotic depletion potential (ADP, el) for minerals and metals (non-fossil resources), the ozone depletion potential (ODP), the photochemical ozone creation potential (POCP), the eutrophication potential (EP), the acidification potential (AP), and the global warming potential (GWP)(The International EPD System, 2022). Additionally, as consumers become more aware of global pollution and start to take into account both the quality of a product and any potential environmental harms, alimentary businesses are focusing more and more on these concerns. The most standard approach for evaluating environmental factors at the moment is life cycle assessment (LCA), which makes it possible to identify and investigate links between the production chain and its impact on natural resources (Del Borghi et al., 2020). As per ISO 14040 (ISO, 2006b) and ISO 14044 (ISO, 2006a), the LCA is a normalized tool that allows the evaluation of the key environmental implications related to a particular good "from the cradle to the grave," by the assessment of multiple input and output streams and their related associated possible environmental consequences on the natural environment. Thus, different analyses were reported in order to determine the potential environmental effects in the production of different types of chocolate: dark, white, and milk.

**Fig. 10:**
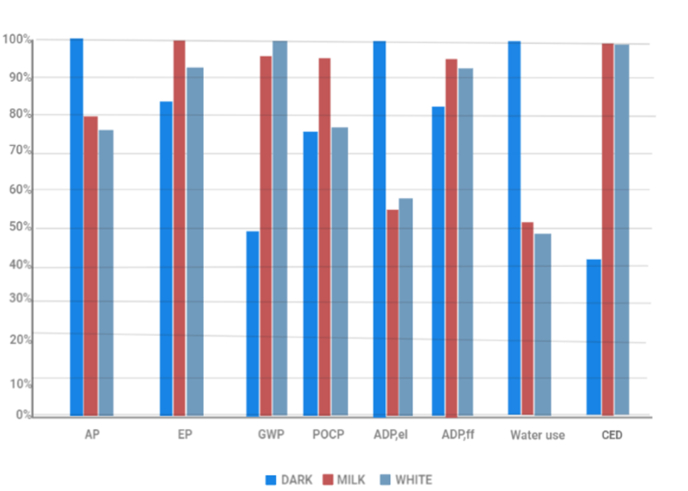
Average component proportions among various producing countries in the world (Ecuador, Ghana, and Indonesia) for dark, milk, and white chocolate are based on assumptions (Bianchi et al., 2021).

As shown in Fig. 10, dark chocolate performs better overall in the areas where the effects of the production on the environment of the manufacturing of milk powder has the biggest impact (i.e., EP, GWP, POCP ADP, ff, CED), whereas milk and white chocolate lose ground as the contribution of milk powder increases. Additionally, because both milk and white chocolate contain the same quantity of milk powder and comparable levels of cocoa co-products, their outcomes are comparable (Table 3). Dark chocolate is regarded by consumers as superior in terms of quality as well as being healthier. Consequently, purchases are anticipated to grow internationally between 2023 and 2028 at a CAGR of 4.1 percent (IMARC, 2022). As a result, despite the fact that similarities between various chocolate varieties fluctuate depending on the type of environmental impact that is taken into account, the production of dark chocolate nonetheless demonstrates the best sustainability impact on the environment, followed by white chocolate and afterward milk chocolate (see Table 5).

## 7 The social responsibility of consumers towards cocoa farmers

Nowadays, as a direct consequence of economic and social shifts and the increasing consumer consciousness, there is less acceptability for business activities that may have a harmful effect on the environment and the lives of local Cocoa farmers. Consumers do not only wish to purchase the best quality on the market, but they also consider certain factors such as the manufacturing procedure, its immediate effects on the environment, and more importantly the labor standards of those involved in the production, product marketing, and promotion or the engagement of the producer in different social efforts. As a result, businesses engage in what is known as “Corporate Social Responsibility (CSR)”(Podsiadły, 2019). Before purchasing, consumers are more attentive to the details found on the product package, and they are especially interested in social or charitable organization symbols that show that a product was produced under certain standards or that a portion of the proceeds from its sale was donated to these organizations. Fairtrade, Rainforest Alliance, and UTZ CERTIFIED are some of the most well-known labels in this area. The first one focuses on different raw materials. Each category of raw materials stipulates both general and specialized requirements. The fully prepared product's certificate indicates, that (Fairtrade International, 2019): farmers earn at least the Fairtrade Minimum Price for their products, which is a set price that is independent of market forces and is calculated to guarantee that the farmer is paid fairly for his or her work, regardless of their gender, workers and farmers are entitled to the freedom of speech. The Rainforest Alliance Certificate was created due to the requirement for the protection of rainforests and biodiversity and is also devoted to various producers. Its appearance on the product indicates that it was manufactured in a way that did not impact the local ecology (The Rainforest Alliance, 2016). Consumers and business partners who see the UTZ Certificate know that the product was procured ethically and sustainably, with no harm done to the environment or those involved in farming and manufacturing (UTZ Certified, 2019). The three certifications described above all call for measures related to corporate social responsibility, although they each emphasize a different component. The Rainforest Alliance focuses on protecting the environment, UTZ CERTIFIED on ensuring the quality of raw materials, and Fairtrade on the condition of farmers and manufacturers. The World Cocoa Foundation, which was founded in 2000, carries out further efforts concerning corporate social responsibility in the processing of cocoa. Currently, the association has more than 100 members, accounting for 80% of the world market for chocolate. The Foundation has taken steps to enhance the living standard of farmers and their families, among other things. Certifications may be adequate evidence for consumers of the social responsibility of manufacturers. Despite this, several large chocolate manufacturers developed their individual CSR programs to promote cocoa production (Table 6).

**Table 5:** The major chocolate producers in Africa.

Company	Country	References
LoshesChocolate	Nigeria	(reogma, 2017), (Alarcón, 2020; Abundanceaware, 2021)
Cocoa Processing Company Limited (CPC)	Ghana	
De Villiers	South Africa	
‘57 Chocolate	Ghana	
Pod Chocolates	Nigeria	
Diogo Vaz	São Tomé	
Lowa Chocolate Factory	Democratic Republic of Congo	
Fairafric	Ghana	
Midunu Chocolates	Ghana	
Afrikoa	South Africa	
MIA	Madagascar	
Cemoi	Ivory Coast	
Beyond Good	Madagascar	
Beyers Chocolates	South Africa	
**Cargill**	Ghana	
**Olam**	Nigeria	
**Kumankoma Company Limited**	Ghana	
**Plot Enterprise Gh**	Ghana	
**Armajaro Ltd**	Ghana	
Chocolatier Robert	Madagascar	
Chocoloza	South Africa

**Table 6:** Material used for chocolate packaging.

Type of material	Type of packaging	Percentage recycled	References
Aluminum foil	Primary packaging	42%	(Miah et al., 2018; Konstantas et al., 2018; Bianchi et al., 2021; Karidis, 2022)
Paper	Primary packaging	87%	
Polypropylene (PP) film	Primary packaging	42%	
Corrugated board components	Secondary packaging	87%	
Polyethylene terephthalate (PET) tray	Primary packaging	42%	
Low-density polyethylene	tertiary packaging	2%	

**Fig. 11:**
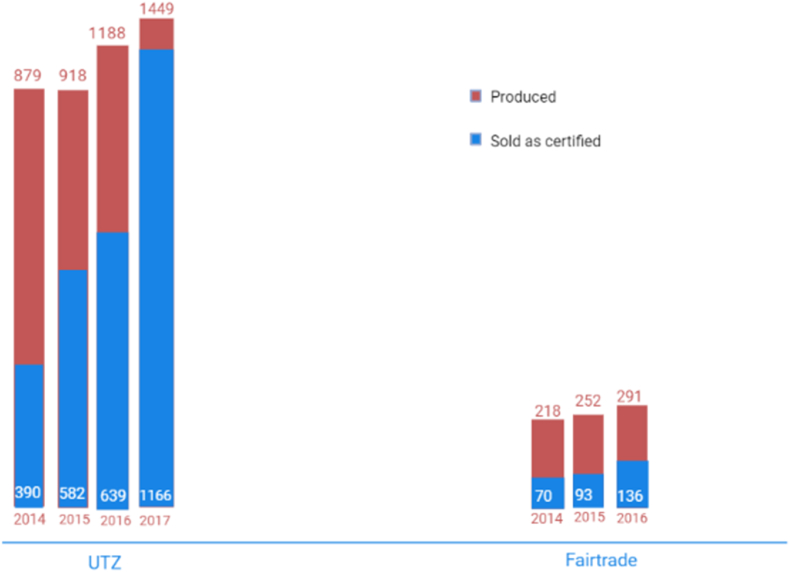
Produced versus sold as a certified chocolate product. (Fountain and Huetz-Adams, 2018).

In comparison to other industries, the cocoa business has made large investments when it comes to corporate social responsibility activities stretching back nearly twenty years. According to the data collected, fair trade farmers have earnings greater than those of uncertificated cocoa. Neither of the norms or initiatives (Table 6) has been able to help farmers considerably achieve a livable wage or even to help them escape persistent misery. The ordinary certified cocoa farmer is still in poverty (Fig. 11) despite a small increase in income among certified workers; the total impression is minimal (Fountain and Huetz-Adams, 2018).

It has been reported that one of the biggest challenges is the use of child labor on cocoa farms, particularly in West Africa (World Wildlife, 2017). Children sometimes forsake formal schooling to work in cocoa orchards, making child labor in the production of cocoa not just a sign of poverty but additionally a contributing cause (Luckstead et al., 2019). Low reserve investment and a shortage of regulating policies are further problems the cocoa industry confronts, leaving African cocoa growers as price-takers (Collins, 2022). More plantations were constructed in different parts of the forest zone by farmers looking to boost their cocoa production. Large-scale deforestation has resulted from each of these searches for additional territory (Wessel and Quist-Wessel, 2015). These difficulties have made it difficult for Africa's chocolate-producing companies (Table 6) to expand and remain sustainable.

Notwithstanding these difficulties, initiatives are being taken to advance the sector. There are continuing conflicts between chocolate manufacturers and local farmers in Ivory Coast, the top producer of cocoa in the world, to better their lives (Collins, 2022). A new generation of chocolate makers from the Ivory Coast is also working to transform the sector and enhance the lives of cocoa growers (Peltier, 2022). In addition, programs are being undertaken to replant cocoa on existing farms to achieve zero child labor and deforestation in the cocoa supply chains (World Wildlife, 2017). Supporting programs for children's education and vocational training is one action that chocolate firms in Africa may take. Child labor rates are lower in areas with better access to high-quality education, according to findings (Weatherill, 2020). In order to lower the percentage of child labor, organizations like the International Cocoa Initiative (ICI) have been trying to increase children's access to education in communities that cultivate cocoa (Sadhu et al., 2020). Also, several chocolate manufacturers have put in place initiatives that provide young people with the skills they need to seek alternative jobs and stay away from risky work (International Labour Organization, 2021).

## 8 The sustainability of the packaging in the chocolate production industry

**Fig. 12:**
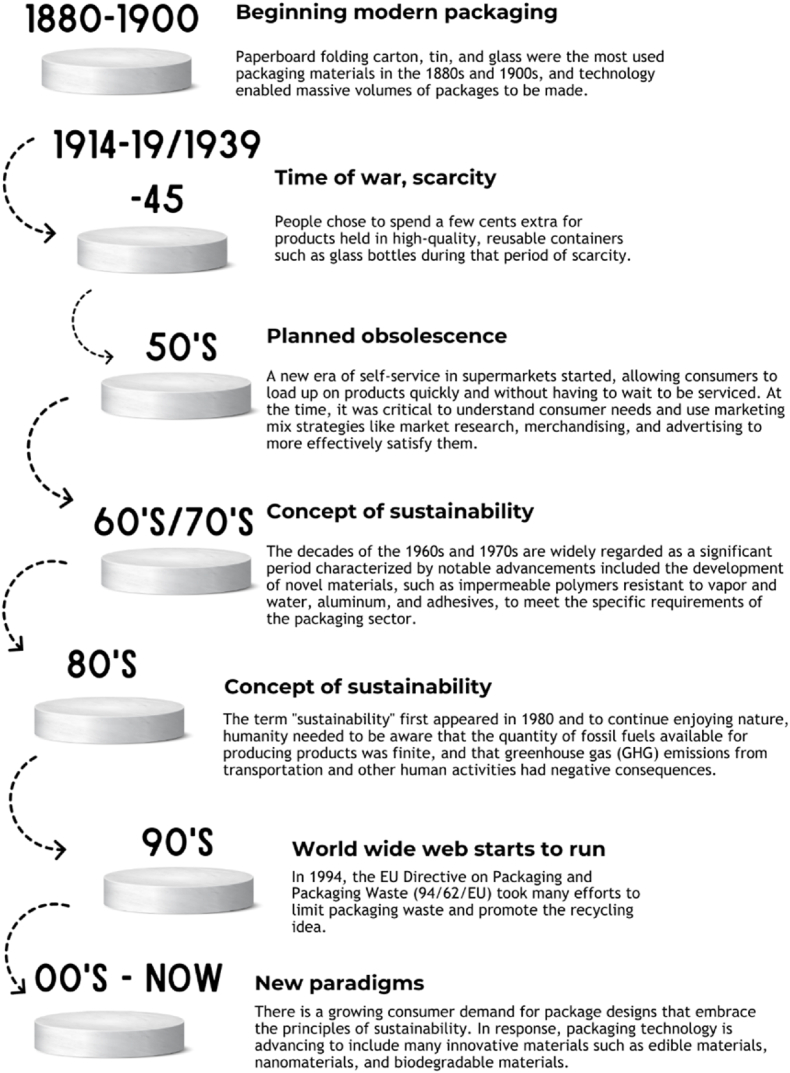
The evolution of food packaging (Escursell et al., 2021).

While additional requirements like economic feasibility and social implications are sometimes overlooked, sustainable packaging is here defined as materials that were obtained sustainably or that can be recovered specifically recyclable or compostable materials, and that have less impact on the environment. Packaging, which comprises designing and establishing procedures to protect the products being sold, is one of the keys to maintaining product quality (Siddiqui et al., 2023). Packaging in the food sector itself has been established since the late 19th century and continues to evolve today. Subsequently, Fig. 12 will succinctly and comprehensively expound on the long history of packaging advancement within the food sector.

Packaging is always designed to communicate the product's message to consumers; today, packaging also serves as a potent marketing tool in addition to protecting the product itself. Designing such packaging is a crucial tactic to assist the rise in product selling volume since consumers are increasingly turning to quick and practical items even though the competition is increasing and becoming more intense in today's market (Lamatinulu et al., 2022). The primary packaging of chocolate products is generally aluminum foil, which is followed by secondary packaging of corrugated cardboard boxes and tertiary packaging of low-density polyethylene (LDPE) film (Konstantas et al., 2018).

Long-term storage of chocolate is typical. The surface might lose its luster and taste fast when in contact with air and light. Because of this, aluminum foil is widely used to provide an obstruction for light, moisture, and other gases, avoiding desiccation, oxidation, and the entry of any unwelcome scent or flavor. The mechanical qualities of aluminum foil also enable the re-wrapping of opened packages, assisting in the reduction of food waste (Büsser and Jungbluth, 2009). The environmental sustainability of cultivation and manufacturing systems is enhanced by choosing materials with less of an influence on the environment. When soy milk is substituted for cow milk, for instance, environmental effects are reduced by 70–99%. However, it is often not possible to replace the materials (Table 6) because the final product's features, like taste, change. So concentrating on the packaging itself is a simple way to lessen the environmental effects of chocolate manufacturing (Hosseinzadeh-Bandbafha and Kiehbadroudinezhad, 2022).

### 8.1 Environmental impact of packaging in the chocolate industry

Similar to other industries, the packaging sector now includes considerations for environmental conservation, social equality, and economic progress. This trend aligns with the prevailing industrial and societal frameworks of the early 21st century. By implementing strategies to enhance the collection, categorization, and processing of packaging materials for recycling, composting, recovery, and waste-to-energy conversion, in addition to adopting efficient disposal and utilization methods for categorized packaging, as well as promoting sustainable sourcing practices and minimizing resource consumption, it is possible to enhance environmentally friendly practices in the packaging supply chain without compromising the essential functions of packaging. The aforementioned concern is notably evident in the context of food packaging composed of polymers. When these materials are inadequately discarded as waste, they give rise to conspicuous environmental pollution in waterways, ultimately leading to contamination in marine ecosystems (Boz et al., 2020). The environmental impact is influenced by the conversion of raw materials into packaging materials and printed designs, which are used for the final packaging of chocolate goods. The environmental indicators that experience an impact include GWP, Water Consumption, ADP, Land Use, and Ecosystem Quality (Miah et al., 2018).

**Fig. 13:**
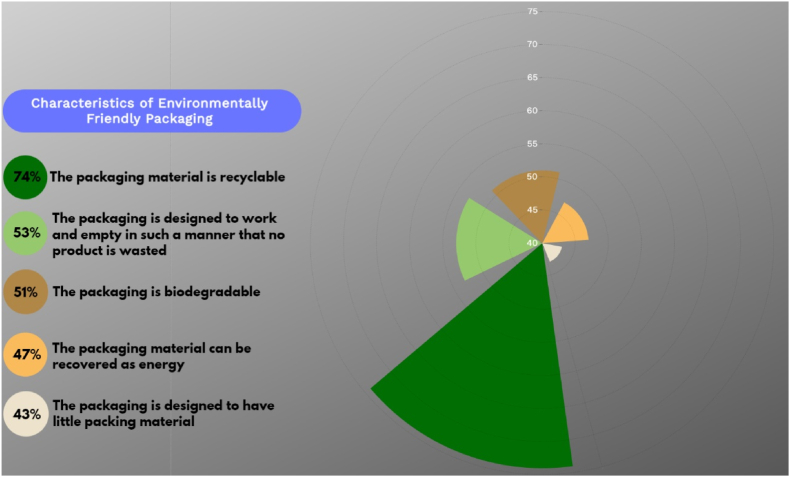
Key characteristics of environmentally friendly packaging for consumers (Korhonen 2012; Otto et al., 2021).

In addition, the growing consumer demand for sustainable packaging has prompted food companies, including those in the chocolate sector, to adopt sustainable manufacturing practices to fulfill market expectations (Korhonen 2012; Otto et al., 2021). Moreover, Fig. 13 provides a more comprehensive elucidation of several attributes of eco-friendly packaging that are often sought after by consumers. In general, consumers have a preference for chocolate product packaging that is recyclable, including materials that include biodegradability and the potential for energy recovery.

#### 8.1.1 Negative environmental impacts of plastic packaging

Materials made of macromolecular polymers are plastics. Most plastics used in wrapping are thermoplastic organic polymers, which contain solely carbon-carbon bonds in their main chains. Examples include polyolefines, polystyrene (PS), and polyvinyl chloride (PVC), although there are also semi-organic polymers like polyamide (PA) and polyesters (PE). In terms of consumption, polystyrene, polypropylene, polyethylene, polyvinyl chloride (PVC), and polyethylene terephthalate, also known as PET, are the five most common packaging polymers. Pallets and agronomic film, bags, coatings, and containers are made of low-density polyethylene (LDPE). While PET is utilized for containers, film, and numerous other food-packaging applications, Polypropylene is used for films, crates, and microwaveable containers. Reusable plastic boxes and trays are replacing disposable cardboard and wooden boxes in secondary and transportation packaging, which also increasingly uses plastic (Kutz, 2007). In terms of global plastic production, plastic packaging utilizes the greatest portion. A little over half of all plastic material is utilized in single-use throwaway products including packaging, agricultural films, and disposable retail products (Hopewell et al., 2009). Large-scale plastic processing, manufacturing, and packaging sectors are frequently sources of industrial refuse (or primary waste). Workshops, artisans, stores, grocery stores, and wholesalers frequently have commercial garbage accessible. These providers will have an extensive range of PE polymers that are frequently contaminated. Outside of metropolitan areas, farms can provide agricultural waste. This often takes the format of packaging or building supplies (Nkwachukwu et al., 2013).

Due to the continuous expansion in plastic material production (along with an ongoing rise in plastic material consumption) in 2013, it was anticipated that the extent of the environmental impact connected with plastic material would rise until 2015. More particular, it was projected that greenhouse gas emissions related to the life cycle of plastic would increase but at a slower rate than in previous decades. In the absence of any new limitations, it could additionally be expected that littering and plastic contamination in marine waterways would have negative implications (Hopewell et al., 2009). It was reported that 96% of the world's packaging materials, for instance, are disposed of in landfills due to a variety of reasons, including but not limited to their easy accessibility (Nkwachukwu et al., 2013).

#### 8.1.2 Environmental impacts of paper

Because plain paper has weak barrier qualities and cannot be heat sealed, it is not recommended to be used to store products for an extended amount of time. Paper is usually treated, covered, layered, or coated with substances like waxes, resins, or lacquers to increase its functional and protective features when used as primary packaging. The types of paper commonly used for chocolate packaging are greaseproof paper, Glassine, and Parchment paper (Marsh and Bugusu, 2007).

On the positive side, paper is a renewable resource, and paper packaging can be recycled multiple times. Additionally, paper packaging can biodegrade and decompose naturally, which reduces the amount of waste in landfills. However, the production of paper can also have negative environmental impacts, such as deforestation and water pollution. Additionally, not all paper packaging is recyclable, and if paper packaging is not properly disposed of, it can contribute to litter and pollution. Chemical usage and the unitary process of making packaging paper are examples of unitary processes that influence the environment. The packaging paper production process is the biggest contributor to environmental impact indicators such as acidification potential (AP) and photochemical ozone creation (POCP). This is a typical occurrence since manufacturing includes key processes and procedures linked to the recovery and processing of recycled paper, the manufacturing of paper, the production of steam, and the processing of wastewater and waste (Iosip et al., 2012).

#### 8.1.3 Environmental impacts of metal packaging

The most adaptable packing material is metal. Superior physical protection and barrier qualities, formability and aesthetic possibilities, recyclability, and consumer acceptability are all included in this product. Aluminum and steel are the two metals that are used in packaging the most frequently. Pure aluminum metal is rolled into extremely thin sheets to create aluminum foil, which may then be folded securely due to it is dead-folding capabilities (a fold produced in the film will remain in place). Additionally, there are many different aluminum foil thicknesses obtainable; thinner foils are used for packaging food, while thicker foils are utilized in trays. similar to all aluminum. Like all aluminum packaging, foil offers a great defense against odors, humidity, air, light, and microorganisms Since it is resistant to acidic meals, lacquer or other forms of protection are not necessary. Even though aluminum is easily recyclable, thin sheets of recovered aluminum cannot be converted into foils without creating pinholes (Marsh and Bugusu, 2007).

Due to the growing use of packaged and processed foods, a greater percentage of packaging materials now makes up the overall quantity of municipal solid waste. To lessen the waste generated by metal packaging material in addition to its negative environmental impacts, several treatments or approaches are used, such as recycling, landfilling, and incineration for energy reuse(Deshwal and Panjagari, 2020). The chemicals used in metal packaging, such as adhesives, coatings, and ornamental paints, are an important cause of hydrocarbon pollution and expose aluminum manufacturing facility employees to cancer-causing polyaromatic hydrocarbons (PAHs) (Pongrácz, 2007). According to research on the environmental impacts of three packaging materials, metalized oriented polypropylene (MOPP) and metalized polyethylene terephthalate (MPET) respectively had a 71% and 52% lower impact on metal depletion than aluminum foil. MOPP and MPET have roughly half the GWP of aluminum foil (Bayus et al., 2016).

#### 8.1.4 Biodegradable and compostable packaging

Biodegradable polymers also referred to as biodegradable plastics (BDPs) are polymeric substances that can decompose into carbon dioxide, methane, water, inorganic elements, or biomass, with the main process being the enzymatic activity of microscopic organisms. The ability to undergo biological breakdown in a compost site as a component of a program, to the extent that the plastic is not identifiable and disintegrates into water, inorganic substances, carbon dioxide, and biomass, at a rate similar to known compostable materials, is required for a subset of BDPs to be considered compostable. Under the right circumstances, the polymer can be degraded by abiotic and biotic processes to a low-molecular-weight molecule. In contrast, the microorganisms must fully use the breakdown products generated or there might be negative effects on the environment and human health (Fornasiero and Graziani, 2006). The outcomes of a process of composting (usually 3 months including an increased temperature >50 °C) must satisfy quality standards such as heavy metal (controlled) concentration, ecotoxicity, and absence of overtly recognizable polymer remnants. For packaging purposes, biodegradable polymers that are functionally and processably similar to conventional petrochemical-based plastic have been designed. These are often created using basic materials that are renewable, such as cellulose or starch. The utilization of sustainable resources (crops as opposed to crude oil) and end-of-life waste disposal through composting or anaerobic digestion to prevent landfilling are the main factors driving demand for biodegradable plastic packaging (Song et al., 2009).

In the chocolate industry, biodegradable and compostable packaging can be made from recycled cardstock, which uses less water to produce and does not require cutting down virgin trees (Nancy, 2021). Innovations in biodegradable and compostable packaging technology are helping to reduce waste and improve sustainability in the chocolate industry. For example, Mars is working to redesign its packaging to be more sustainable and plans to switch to paper packaging for its Snickers bars. Other companies, such as Cacoco, are already using 100% compostable packaging for their products (Cecchini, 2017). These innovations demonstrate the potential for biodegradable and compostable packaging to become the norm in the chocolate industry and beyond, leading to a more sustainable future.

### 8.2 Consumer buying behavior towards sustainable packaging

The number of research assessing the impact of sustainable packaging on consumers' buying behaviors is quite low, although there are numerous studies on consumer opinions toward package design components. This lack of information with a clear direction may account for why sustainable packaging frequently falls short of market expectations while being heavily advertised. Furthermore, people may not want to transition from their favorite items to sustainable innovative packaging if it fails to include the product they prefer (Boz et al., 2020). Different studies show that the packaging is especially important because chocolate is frequently bought as a gift. Thus, the chocolate's quality and the packaging it comes with are equal in importance. The product that has the most appealing packaging is generally chosen by the consumer if they are unfamiliar with the product (Del Prete and Samoggia, 2020a,b). Many startups are taking the initiative to implement sustainable chocolate packaging in their businesses. One way they are doing this is by using biodegradable materials for their packaging. For example, Alter Eco, a food brand, has found sustainable alternatives to non-recyclable plastic used for chocolate truffle packaging. Other startups researching sustainable packaging trends that are plastic-free include Tony's Chocolonely, Vegan Galaxy, Divine Chocolate, Happi Free From, and Conscious Chocolate (Caroline, 2021).

According to a study, the package message will have a larger influence on the buyer if the information on the packaging is written in a specific size and manner (Rebollar et al., 2015). Another study found that sustainable packaging does not have an additive effect on the perceived product quality of chocolate (Mai, 2014). The consumer's desire to purchase a particular chocolate is additionally affected by the packaging of the chocolate (Beneke et al., 2015). The use of attractive packaging can help draw consumers towards a particular chocolate brand and increase the likelihood of purchase. The packaging design can be used to highlight key features of the product, such as the flavor, texture, and ingredients, and entice the consumer to try it (Lau, 2022). A comparative analysis of various brand names such as Nestle appearing on the packaging of chocolate influences the buying behavior of the consumers by the dominance of its respective category (Laforet, 2011). It was also reported that consumers normally link the brand name Cadburys with the color purple. Since consumers might associate even a simple purple package with Cadbury's, the color alone can create a brand identity. This study also revealed that the use of appealing colors in chocolate packaging has a favorable effect on consumers' buying behavior (Arya and DineshBabu, 2021). Another comparative study shows that emotional factors like nostalgia affected the purchasing behavior of chocolate brands like Kit-Kat and Milk Silk (Dhar and Shil, 2022).

## 9 Conclusion

Consumer buying behavior is becoming an important subject as a result of its significant effects and concurrently, increased consumer focus on choosing more sustainable product alternatives. The objective of this review was to determine whether the practices followed in the chocolate production processes throughout the food supply chain affect consumer buying behavior. It was shown that following the value chain of dark, white, and milk chocolate and considering their life cycle in several locations that manufacture cocoa, the consumer buying behavior was directed to dark chocolate which was the most sustainable product in comparison to milk, and white chocolate. Consumer buying behavior could also be influenced by the socio-economic life condition of local farmers which is improved under the umbrella of certifications and corporate social responsibility initiatives. It was also seen that the packaging can also influence the consumers’ perception through the different features that it can have such as the name of the brand, and its color. To attain more sustainable cocoa, companies should be conscious of setting standards that will incorporate living income as one of the most important areas of corporate business responsibility and also help minimize deforestation related to cocoa farming by only processing cocoa that has been harvested from reused land. Although certification can help businesses improve their value chain, much more will be needed to become sustainable, involving collaboration with other businesses and significant government participation. To successfully get a livable wage, innovative strategies are required. Until dedicated workers can earn a liveable wage and more care is given to the impact that the production process of chocolate can have on the environment, cocoa cultivation cannot be sustained. The industry has to adopt net worker income as an indicator, and information about it needs to be published.

This study also could provide comprehensive guidelines for further research development to maximize consumer responsibilities in promoting sustainable cocoa production. For instance, long-term studies would potentially look at how consumer preferences and purchase behavior are influenced by lifecycle assessments of various chocolate varieties over time; it could also examine the environmental effects at every step through production and how consumers are informed about them. Moreover, it is important to investigate further the link between consumer awareness of the socioeconomic circumstances of local farmers and their purchasing choices, paying particular attention to the efficacy of corporate social responsibility and certification programs. Further research is also necessary on the particular aspects of chocolate packaging, such as brand name, color, and sustainability claims, that affect consumer perceptions. Additionally, research should be addressed on how government regulations affect consumer behavior and how they support sustainable cocoa production. Innovative business approaches and strategies aimed at ensuring livable wages for cocoa workers should be investigated, evaluating their viability and effect on consumer advocacy and industry sustainability. This all-encompassing strategy will provide insightful information and guidance on the complex relationships that exist among consumer behavior, business practices, and sustainability in the cocoa sector.

## Funding information

The open-access publishing fee is covered under the agreement between Elsevier and the Hungarian consortium EISZ upon acceptance due to Gyula Kasza and Widya Satya Nugraha being affiliated with the 10.13039/100020610Hungarian University of Agriculture and Life Sciences.

## CRediT authorship contribution statement

**Shahida Anusha Siddiqui:** Conceptualization, Methodology, Validation, Formal analysis, Writing – original draft, Writing – review & editing, Visualization, Data curation, Investigation, Supervision. **Ikawati Karim:** Writing – original draft. **Chardi Shahiya:** Writing – original draft, Visualization. **Sergey Shityakov:** Review. **Widya Satya Nugraha:** Writing – review & editing, Visualization, Resources, Supervision. **Gyula Kasza:** Writing – review & editing, Resources, Supervision.

## Declaration of competing interest

The authors declare no conflict of interest.

## Data Availability

Data will be made available on request.
